# Telemedicine for the Management of Glycemic Control and Clinical Outcomes of Type 1 Diabetes Mellitus: A Systematic Review and Meta-Analysis of Randomized Controlled Studies

**DOI:** 10.3389/fphar.2017.00330

**Published:** 2017-05-30

**Authors:** Shaun W. H. Lee, Leanne Ooi, Yin K. Lai

**Affiliations:** ^1^School of Pharmacy, Monash University MalaysiaBandar Sunway, Malaysia; ^2^Faculty of Pharmacy, UCSI UniversityKuala Lumpur, Malaysia

**Keywords:** telemedicine, type 1 diabetes, management, meta-analysis, systematic review

## Abstract

**Importance:** Telemedicine has been shown to be an efficient and effective means of providing care to patients with chronic disease especially in remote and undeserved regions, by improving access to care and reduce healthcare cost. However, the evidence surrounding its applicability in type 1 diabetes remains scarce and conflicting.

**Objective:** To synthesize evidence and quantify the effectiveness of telemedicine interventions for the management of glycemic and clinical outcomes in type 1 diabetes patients, relative to comparator conditions.

**Data Sources:** MEDLINE, EMBASE, Cochrane Library, Web of Science, PsycINFO, and CINAHL were searched for published articles since inception until December 2016.

**Study Selection:** Original articles reporting the results of randomized controlled studies on the effectiveness of telemedicine in people with type 1 diabetes were included.

**Data Extraction and Synthesis:** Two reviewers independently extracted data, assessed quality, and strength of evidence. Interventions were categorized based upon the telemedicine focus (monitoring, education, consultation, case-management, and peer mentoring).

**Main Outcome and Measure:** Absolute change in glycosylated hemoglobin A1c (HbA1c) from baseline to follow-up assessment.

**Results:** A total of 38 studies described in 41 articles were identified. Positive effects on glycemic control were noted with studies examining telemedicine, with a mean reduction of 0.18% at the end of intervention. Studies with longer duration (>6 months) who had recruited patients with a higher baseline HbA1c (≥9%) were associated with larger effects. Telemedicine interventions that involve individualized assessments, audit with feedback and skill building were also more effective in improving glycemic control. However, no benefits were observed on blood pressure, lipids, weight, quality of life, and adverse events.

**Conclusions and Relevance:** There is insufficient evidence to support telemedicine use for glycemic control and other clinically relevant outcome among patients with type 1 diabetes.

## Introduction

The prevalence of type 1 diabetes has been steadily increasing for the past few decades (You and Henneberg, 2016). In many of these patients, diabetes-related complications are the major cause of morbidity and mortality. The Diabetes Control and Complications Trial (DCCT) showed that tight glycemic control reduces the risk of complications and progression of microvascular complications (nephropathy, neuropathy, and retinopathy) in patients with type 1 diabetes (The Diabetes Control Complications Trial Research Group, 1993). The use of technological innovation in routine practice represents an attractive option for improving outcomes in these patients as several studies have demonstrated the feasibility and acceptability of using telemedicine (Farmer et al., 2005; Charpentier et al., 2011; Lee et al., 2015, 2016) in diabetes management.

Recognizing the importance and value that technology can bring in providing individualized health care especially for chronic diseases, the Agency for Healthcare Research and Quality recently commission a technical report to determine the impact of telehealth on healthcare (Totten et al., 2016). The report found that telemedicine was beneficial for patients with chronic conditions. Several reviews have similarly documented the potential of telemedicine in the management of diabetes, but the evidence is relatively scarce for type 1 compared to type 2 diabetes. Furthermore, findings from several reviews have been mixed thus far. We therefore conducted a systematic review and meta-analysis to determine the potential of telemedicine use in the management of type 1 diabetes patients.

## Methods

### Study Eligibility Criteria

We searched six databases and gray literature from inception until December 2016 without any language restriction using the search terms as listed in Supplement and PROSPERO (CRD42016053117). This was supplemented by a hand search of the reference list of retrieved articles and relevant systematic reviews. The search results and full-text articles were screened independently by two separate reviewers (YL and LO) to determine relevance and any disagreement resolved through consensus or adjudication by a third reviewer (SL). All randomized controlled trials (RCTs) were included if they examined the use of telemedicine in patients with type 1 diabetes with a comparator (usual care or active comparators). Telemedicine was operationally defined as the use of medical information exchanged from one site to another via electronic communication (fax, short message system, internet, modem, telephone, mobile phone or its applications) to improve a patient’s clinical health status.

### Data Extraction and Quality Assessment

Data on baseline characteristics and intervention details as well as other relevant outcomes [e.g., hemoglobin A1c (HbA1c), blood pressure] were independently extracted by two reviewers (LO/YL and a research assistant). Additional information was obtained from the corresponding author or published protocols when available. All data were double-checked by a third reviewer (SL) for accuracy before analysis. The primary efficacy measure was the mean change in absolute glycated hemoglobin. Other outcomes include changes in hypoglycemia rates, blood pressure, body weight, self-management, and adverse events. All studies were evaluated for quality assessment using the Cochrane Risk of Bias tool (Higgins et al., 2011) and the quality of each evidence was assessed using the GRADE approach (Schünemann et al., 2008).

### Intervention Classification

Intervention and control conditions were classified into either one of the following categories based upon an adaptation of the definitions from the American Telemedicine Association (2016):

Teleeducation: Any intervention aimed at educating, teaching, or training patients remotely using live interactive streaming or by stored educational material.
Telemonitoring: Any process which allows for the delivery and/or exchange of information to monitor a health status of a patient remotely.
Teleconsultation: Two-way communication between a patient and healthcare provider aimed at providing care from a distance.
Telecase-management: Any collaborative initiative to integrate the assessment, care coordination, or evaluation to meet the needs of the patient.
Telementoring: Any intervention to support, guide, or mentor an individual from a distance by another peer who has gone through a similar experience

### Statistical Analysis

We determined the difference between baseline and post-intervention values for each comparison. In the event of missing information, data was imputed from baseline values, *p*-values, 95% confidence intervals or interquartile range using a correlation of 0.5. We subsequently performed a random-effects pairwise meta-analysis using the mean difference (MD) for continuous data or odds ratio for dichotomous data when at least two studies examined the same intervention and comparator for an outcome. Study heterogeneity was determined using the *I*^2^ statistics. To determine the robustness of our results, we performed sensitivity analyses by using alternative effect measures. Publication bias was assessed visually and quantitatively using the Egger test for comparison with a minimum of 10 studies. We also examined subgroup analyses to determine if a particular telemedicine strategy was more or less effective in reducing HbA1c based on population characteristics as well as intervention characteristics. Univariable weighted linear regression analyses were performed to evaluate for effect modification on HbA1c at the end of intervention. All statistical testing was two-sides, with a *p*-value of <0.05 considered statistical significant using Stata 13.0 (Stata Corp).

## Results

### Study Characteristics

The initial search yielded 422 articles and 41 articles from 38 unique studies involving 2,582 participants were identified (Supplementary Figure 1). Thirty-three studies were parallel group RCTs, four were crossover studies and one study was a waitlist RCT. Most studies were single center, conducted in Europe or North America. At baseline, the proportion of female participants ranged between 32 and 100% (in studies examining pregnant women). Eighteen studies were conducted in children and adolescents (mean age: 14 years, range: 9.9–16.8 years) while another 19 studies were conducted in adults (mean age: 24.3 years, range: 23.9–42.9 years). The mean baseline HbA1c ranged from 6.75 to 10.15% and duration of type 1 diabetes from 5.7 to 18.9 years. Sample size of studies ranged from 10 to 180, with the length of intervention ranging from 2 weeks to 12 months (Supplementary Table 1).

### Intervention Characteristics

The telemedicine system used in most trials were relatively simple and involved data transmission of blood glucose data with feedback (*n* = 19; Ahring et al., 1992; Marrero et al., 1995; Fallucca et al., 1996; Biermann et al., 1999; Gómez et al., 2002; Chase et al., 2003; Montori et al., 2004; Vähätalo et al., 2004; Farmer et al., 2005; Lawson et al., 2005; Rami et al., 2006; Benhamou et al., 2007; Cadario et al., 2007; Rigla et al., 2008; Landau et al., 2012; DeSalvo et al., 2013; Kirwan et al., 2013; Berndt et al., 2014; Shalitin et al., 2014; Schiaffini et al., 2016) or blood glucose data only (*n* = 2; Wojcicki et al., 2001; Chase et al., 2003). Data was mostly transmitted daily in five trials, weekly or less often in 15 trials and in one studies the timing was unspecified. The methods for providing feedback include text messages, phone calls as well as standardized messages. In four trials, the authors had used web-based educational modules (Rossi et al., 2010, 2013; Newton and Ashley, 2013; Harris et al., 2015) and another five studies used telephone (Howells et al., 2002; Panagiotopoulos et al., 2003; Nunn et al., 2006; Lehmkuhl et al., 2010), or SMS (Louch et al., 2013) to provide education to their patients. Five studies had used teleconferences (Gay et al., 2006; Jansà et al., 2006; Izquierdo et al., 2009; Charpentier et al., 2011; Esmatjes et al., 2014) to conduct remote clinic visits. Two studies had used a case manager to help assist individuals cope with their condition (Howe et al., 2005; McCarrier et al., 2009) while one study had a peer-mentor to share their experience and support the patient (Suh et al., 2014). All these studies primarily used telephone, mobile phones, modem, or internet to transmit their data. Only one study also used fax as a mode of transmission (Supplementary Tables 2–5).

### Risk of Bias

Using the Cochrane risk of Bias tool, 33 trials had an unclear risk of bias in the masking of participants, investigators, or both domain (86.8%); detection bias in 31 trials (81.6%); allocation concealment in 23 trials (60.5%); random sequence generation 14 trials (36.8%); incomplete outcome domain in five trials (13.2%); and in three trials incomplete outcome reporting (7.9 %). Eight studies had a high risk of bias in one or more of the domains assessed (Supplementary Figure 2).

### Effectiveness of Telemedicine: Key Outcomes

Twenty-eight studies examining 2,099 participants were included in the meta-analysis. Overall, telemedicine was found to reduce HbA1c by 0.18% (95% CI: 0.04–0.33; *p* = 0.01) at the end of the intervention, but a high level of heterogeneity was observed (66.1%, *p* ≤ 0.01). Inspection of the effect size identified two studies (Charpentier et al., 2011; Kirwan et al., 2013) with effect sizes larger than other trials. Exclusion of these two studies removed heterogeneity, and reduced the impact of telemedicine (-0.01%, -0.05 to 0.02; *I*^2^ = 0%). No significant improvements were noted at the end of 3 or 6 months follow-up (Supplementary Figure 3). **Table 1** summarizes the findings for all the key outcomes in comparison of telemedicine use in type 1 diabetes studies.

**Table 1:** Summary estimates and strength of evidence for key outcomes of telemedicine studies compared to comparator in type 1 diabetes.

Outcomes assessed	Outcome timing	Total participants	No of studies	Study effect (95% CI)	*p*-value	*I*^2^ statistics (%)	Strength of evidence
HbA1c (%)	EOI	2099	28	MD: -0.18 (-0.33 to -0.03)	0.01	66	Very low
Adults		1256	15	MD: -0.26 (-0.47 to -0.05)	<0.01	80	
Children and adolescent		796	11	MD: -0.12 (-0.30 to 0.05)	0.70	0	
Pregnant mother		47	2	MD: 0.35 (-0.13 to 0.84)	0.77	0	
HbA1c (%)	3-month follow-up	143	2	MD: -0.50 (-1.89 to 0.89)	0.48	89	Very low
HbA1c (%)	6-month follow-up	85	2	MD: 0.15 (-0.54 to 0.84)	0.67	84	Very low
Fasting plasma glucose (mmol/L)	EOI	324	4	MD: -0.34 (-2.24 to 1.56)	0.73	90	Very low
Body mass index (kg/m^2^)	EOI	258	4	MD: -0.04 (-2.31 to 2.24)	0.98	81	Very low
Systolic blood pressure (mmHg)	EOI	242	2	MD: 0.45 (-2.04 to 2.94)	0.72	58	Very low
Diastolic blood pressure (mmHg)	EOI	242	2	MD: -1.57 (-3.33 to 0.20)	0.08	59	Very low
Total cholesterol (mmol/L)	EOI	242	2	MD: 0.36 (-9.78 to 10.49)	0.95	74	Very low
Low-density lipoprotein (mmol/L)	EOI	242	2	MD: 1.49 (-4.33 to 7.31)	0.62	47	Very low
High-density lipoprotein (mmol/L)	EOI	242	2	MD: -0.85 (-4.75 to 3.06)	0.67	83	Very low
Triglycerides (mmol/L)	EOI	242	2	MD: -4.64 (-29.48 to 20.19)	0.71	88	Very low
Severe hypoglycemia	EOI	905	13	OR: 0.82 (0.35–1.75)	0.61	0	Low
Diabetic ketoacidosis	EOI	440	8	OR: 0.86 (0.31–2.37)	0.77	0	Low

*EOI, end of intervention; HbA1c, hemoglobin A1c; MD, mean difference; OR, odds ratio*.

**FIGURE 1:**
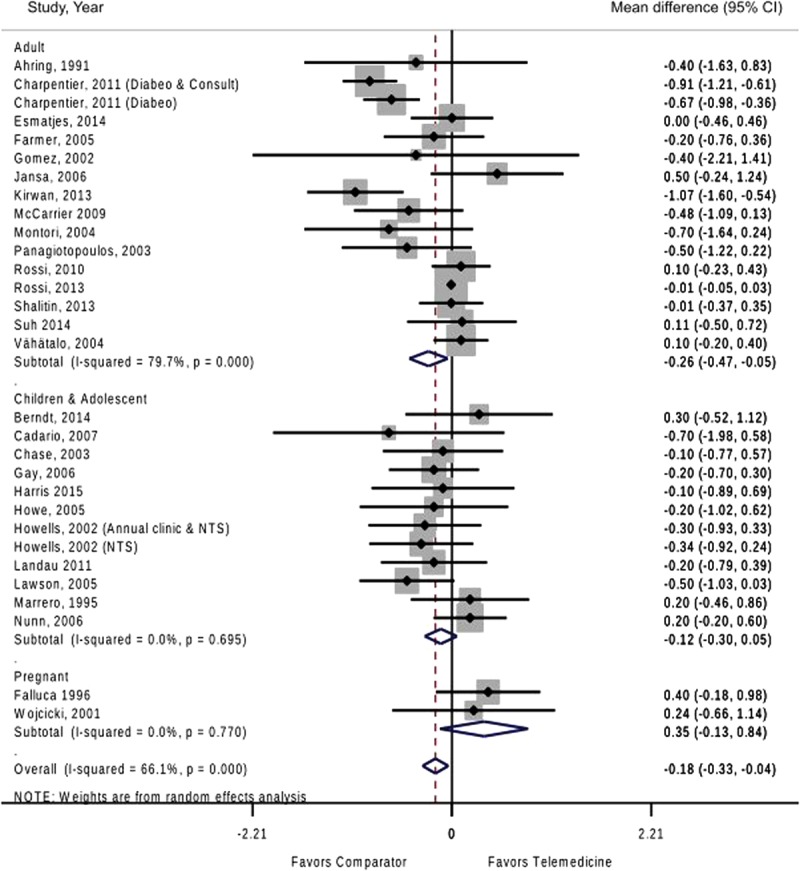
Effect of telemedicine versus comparator on hemoglobin A1c at end of intervention.

Subgroup analyses of results showed that adults appear to benefit more than adolescents, children, or pregnant mothers at the end of study (**Figure 1**). Effectiveness of telemedicine was higher when the studies were at least 6 months in duration or longer, in studies conducted in North America as well as those who had recruited participants with a higher baseline HbA1c of ≥9% (Supplementary Table 6). Analysis by type of telemedicine activity showed that only teleeducation and teleconsultation had a positive effect in improving glycemic control. No difference were noted between studies with an active control compared to usual care (-0.17% versus -0.15%; Supplementary Figure 4). Visual inspection of funnel plot based on all 26 studies showed mild form of asymmetry, suggesting the presence of publication bias (more small studies favoring telemedicine; Supplementary Figure 5), but estimate was not significant (Egger test: bias -0.68; *p* = 0.06).

When considering other efficacy outcomes, no significant difference were noted with the use of telemedicine in reducing blood pressure, lipid levels, body mass index as well as fasting plasma glucose (Supplementary Figures 6–13). In the 11 trials that reported quality of life (Gómez et al., 2002; Lawson et al., 2005; Jansà et al., 2006; Benhamou et al., 2007; Rossi et al., 2010, 2013; Charpentier et al., 2011; Kirwan et al., 2013; Esmatjes et al., 2014; Shalitin et al., 2014; Suh et al., 2014), no differences were reported between telemedicine and usual care when using generic health-related quality of life (SF12, EuroQoL) or diabetes specific quality of life (Diabetes Quality of Life for Youth, Diabetes Quality of Life).

The cost-effectiveness of telemedicine intervention was unclear as four studies (Chase et al., 2003; Jansà et al., 2006; Charpentier et al., 2011; Esmatjes et al., 2014) reported lower cost in the telemedicine group while another four studies reported higher cost (Biermann et al., 1999; Nunn et al., 2006; Rossi et al., 2010; Kirwan et al., 2013). In one study, the cost implications of telemedicine was unclear (Benhamou et al., 2007) while another study reported similar cost between telemedicine and usual care (Cadario et al., 2007). No significant difference between telemedicine and usual care for odds of severe hypoglycemia (Supplementary Figure 14) or diabetes ketoacidosis (Supplementary Figure 15) were reported. No studies reported on diabetes-related complications, healthcare utilization, or harms outcome.

### Effectiveness on Glycemic Control in Subpopulations

Based upon our subgroup analysis, adults appear to be benefit from activities that provided education and consultation (Supplementary Figure 16). These activities appear most effective when the content was delivered using a mobile phone compared to other modalities. In adolescents, a similar trend was noted, with results from telemonitoring studies reaching statistical significance (MD: -0.32%; -0.65% to 0.00; Supplementary Figure 17). Similarly, studies using telephone as a delivery mechanism was the most effective in this group. The small number of studies for children and pregnant women limited our ability to draw any conclusions.

### Moderation of Program Content and Mechanisms on Effectiveness

Analysis of study features found that studies which incorporated elements of individualized assessment, audit with feedback, skill building and theory based counseling as well as high intensity intervention (weekly or more often contacts delivered over a longer duration of 6 months) were positively associated with higher improvements and thus more likely to associated with higher success in type 1 diabetes individuals (Supplementary Table 6). Univariable meta-regression analyses to assess whether treatment effects for HbA1c were modified by population and intervention characteristics showed that all parameters assessed had little to no effects on the outcomes assessed. The lack of reporting on other outcomes such as ethnicity or socioeconomic status precluded analysis of other parameters in our analysis.

## Discussion

The evidence on use of technology in diabetes has been evolving, with mixed results (Supplementary Table 7). Results of this study suggest that telemedicine may have a role in the glycemic management of type 1 diabetes patients, particularly in adults. Nevertheless, our results must be interpreted with caution due to the high level of heterogeneity and lack of significance when the sources of heterogeneity were excluded. The evidence for supporting the use of telemedicine in other outcomes such as blood pressure, self-management, and quality of life is limited. In terms of adverse outcomes, we noted potential clinically important but non-statistically significant reduction in the episodes of severe hypoglycemia and diabetic ketoacidosis. Our subgroup analysis found that the effectiveness of telemedicine strategies varied depending on the target population. For example, we noted that telemedicine strategies that intervened on patient educating and/or monitoring were associated with the largest treatment effects among adults and adolescents. Our findings also suggest that telemedicine interventions that used mobile phones were associated with the largest effects.

Results also indicate for the telemedicine intervention that to be successful, it would need to incorporate elements of skill building aimed at improving diabetes knowledge. The intervention would also need to have a high degree of responsiveness to an individual’s need and conducted over a period of not less than 6 months. Our review also identified 10 studies which reported on the cost savings and financial gains of telemedicine. However, results have been conflicting, making it difficult for us to draw any conclusion regarding this outcome. This was confounded by the wide range of methodology for assessing the cost-effectiveness of intervention.

Nevertheless, the potential for telemedicine to reduce the number of clinic visits while maintaining glycemic control is clinically desirable and potentially cost-effective. Thus, more studies should be conducted to determine the cost-utility of telemedicine, return of investment and what aspect of diabetes care that could be potentially replaced by telemedicine. Future studies can also explore the role of telemedicine for monitoring especially among adolescent, as this would represent a unique opportunity for parents to provide their children with some autonomy and independence in their own diabetes management.

Our findings has potential ramifications in terms of practice and policy. For example, the UKPDS study have shown that a 1% reduction in mean HbA1c has been associated with a 21% reduction in diabetes-related death and 37% reduction in microvascular complications in type 2 diabetes patients (Stratton et al., 2000). If telemedicine was implemented in all type 1 diabetes patients, this may potentially translate to a 3.8% reduction in diabetes-related death and 6.7% fewer microvascular complications. The fear of hypoglycemia has been associated with various self-management strategies including reduction in insulin use and increased energy intake. As telemedicine could reduce the risk of severe hypoglycemia by up to 18%, this could help patients achieve improved glucose control for longer and reduce the risk of microvascular and macrovascular complications.

Our systematic review has some important limitations. Our review identified only four studies examining teleconsultation and telecase-management programs, which limited our ability to draw conclusions about the difference in benefits. The limited evidence base also did not allow us to make firm conclusions for many of the outcomes assessed. The diversity of definitions of telemedicine which included devices such as telephone, modems, mobile phones, and websites made the assessment of treatment efficacy and cost-effectiveness difficult. Most studies had a lack of reporting on the blinding of participants and outcome personnel as well as intention to treat analysis. The definition of usual care was inconsistent across studies, as 13 studies had an enhanced usual care (including reminder telephone calls) during this study. In some studies, a less intensive form of telemedicine was used. This may have reduced the relative effectiveness of telemedicine if implemented in routine clinical practice. The use of GRADE system (Supplementary Table 8) also resulted in most evidence being classified as either low or very low, and thus underestimates the effects.

Despite the modest improvement in HbA1c, several aspects of telemedicine warrants investigations in future studies. For example, it is important to determine whether the effects of telemedicine differ on the administrator, type, frequency and mode of data transmission as well as the target participant. In addition, most studies should also measure other patient important outcomes such as quality of life, cost-effectiveness, patient satisfaction, and acceptance. As such, it is difficult to determine the true value of this technology based intervention or determine the type of technology that is most beneficial. Future studies should build explicitly upon the present evidence base and target a broad range of important diabetes outcome, carefully assessing the role of telemedicine with a longer term follow-up to determine the sustainability of intervention. These telemedicine interventions should also be thoroughly described.

In summary, results of this study provide a reason for optimism that telemedicine intervention can be effective in type 1 diabetes patients. It highlights the important intervention components needed to improve diabetes care, including individualized assessments targeted at improving knowledge and use behavioral methods with sufficiently high intensity (weekly contact or more frequent) over a long duration (≥6 months). However, further research is needed to determine which strategy is the most effective in different patient subgroups, and the effects on long-term effects clinical outcomes, that incorporate a formal economic analysis.

## Author Contributions

SL had full access to all the data in the study and is accountable for all aspects of the work, in ensuring that questions related to accuracy or integrity of any part of the work are appropriately investigated and resolved. SL and YL contributed to study concept, design, and drafting of manuscript. All authors involved in acquisition, analysis, or interpretation of data and critical revision of the manuscript for important intellectual content. SL performed statistical analysis and supervised the study. All authors read and approved the final manuscript.

## Conflict of Interest Statement

The authors declare that the research was conducted in the absence of any commercial or financial relationships that could be construed as a potential conflict of interest. The reviewer AC declared a shared affiliation, though no other collaboration, with two of the authors SL, LO to the handling Editor, who ensured that the process nevertheless met the standards of a fair and objective review.
