# Supplementary material for: Telemedicine for the Management of Glycemic Control and Clinical Outcomes of Type 1 Diabetes Mellitus: A Systematic Review and Meta-Analysis of Randomized Controlled Studies
Source: Front Pharmacol. 2017 May 30;8:330. doi: 10.3389/fphar.2017.00330 (PMC5447671; doi:10.3389/fphar.2017.00330)
Supplement: Supplementary file 1 [file Table_1.PDF]

Supplementary Material

Telemedicine for the management of glycemic control and clinical outcomes of type 1 diabetes mellitus: A systematic review and meta-analysis of randomized controlled studies

Shaun Wen Huey Lee*, Ooi Leanne, Yin Key Lai,

* Correspondence: Corresponding author: shaun.lee@monash.edu

Search strategy used in the current study

We searched MEDLINE using the algorithm listed below. The algorithm was adjusted to also search the Cochrane Library,EMBASE, PsycINFO, Web of Science and CINAHL databases.

Database: Ovid MEDLINE® <1970 to December Week 4 2016> Search Strategy:

1. exp diabetes, type 1
2. T1D.mp
3. Type 1 diabetes.mp
4. #1 OR #2 OR #3
5. Telehealth.mp
6. telemedicine.mp
7. telemonitoring.mp
8. telecare.mp
9. telematics.mp
10. telehomecare.mp
11. mobile health.mp
12. remote monitoring.mp
13. #5 OR #6 OR #7 OR #8 OR #9 OR #10 OR #11 OR #12
14. remote consultation.mp
15. sms.mp
16. short messaging system.mp
17. video monitoring.mp
18. internet monitoring.mp
19. internet consultation.mp
20. video consultation.mp
21. telephone.mp
22. smartphone.mp
23. cellular phone.mp
24. mobile phone.mp
25. telephone support.mp
26. #14 OR #15 OR #16 OR #17 OR #18 OR #19 OR #20 OR #21 OR #22 OR #23 OR #24 OR #25 OR #26
27. #26 OR # #13
28. #27 AND #4

Supplementary Figure 1: Summary of evidence search and selection through the review process

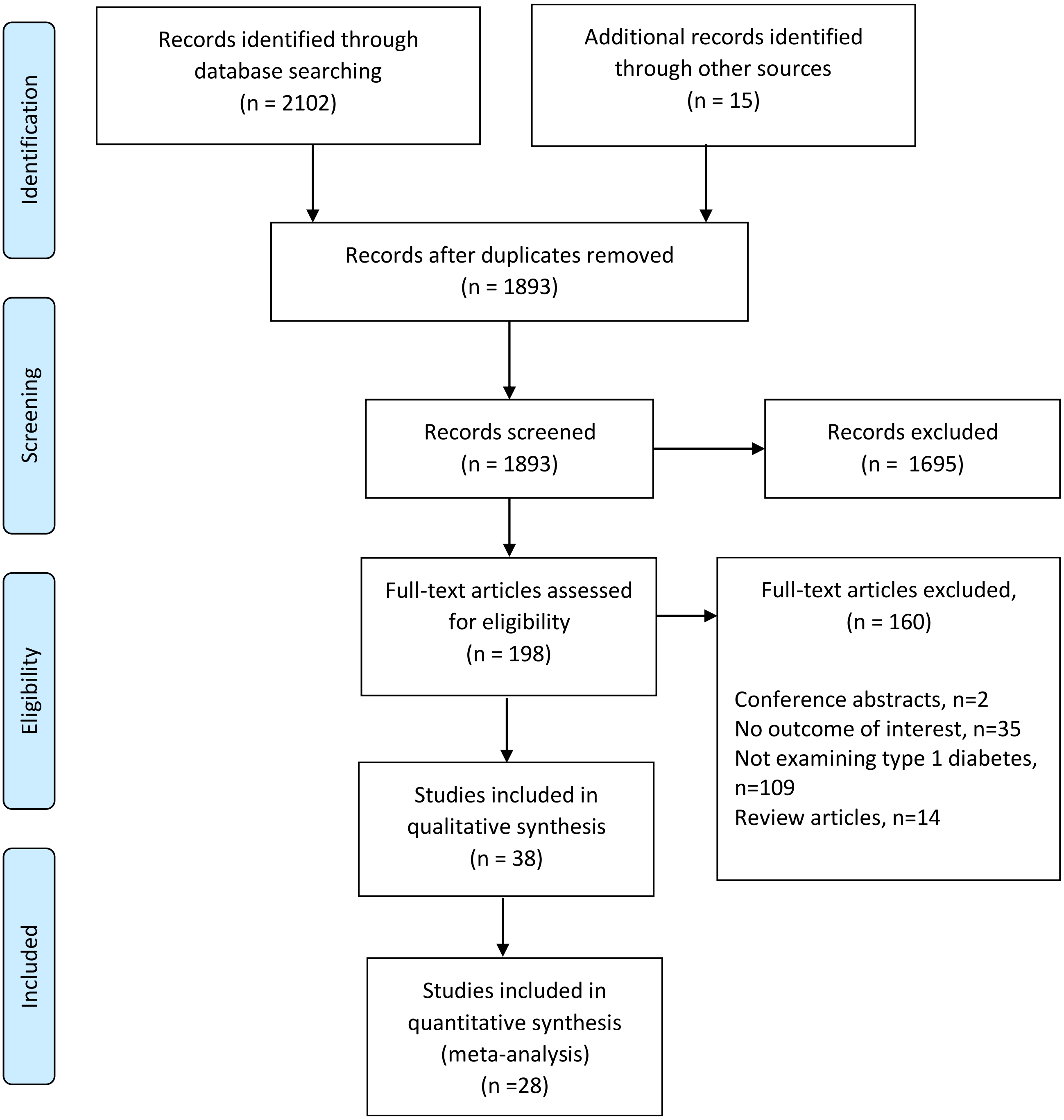

Supplementary Figure 2: Risk of bias of included trials, evaluated using Cochrane risk of bias tool based upon reviewers' judgement of each domain

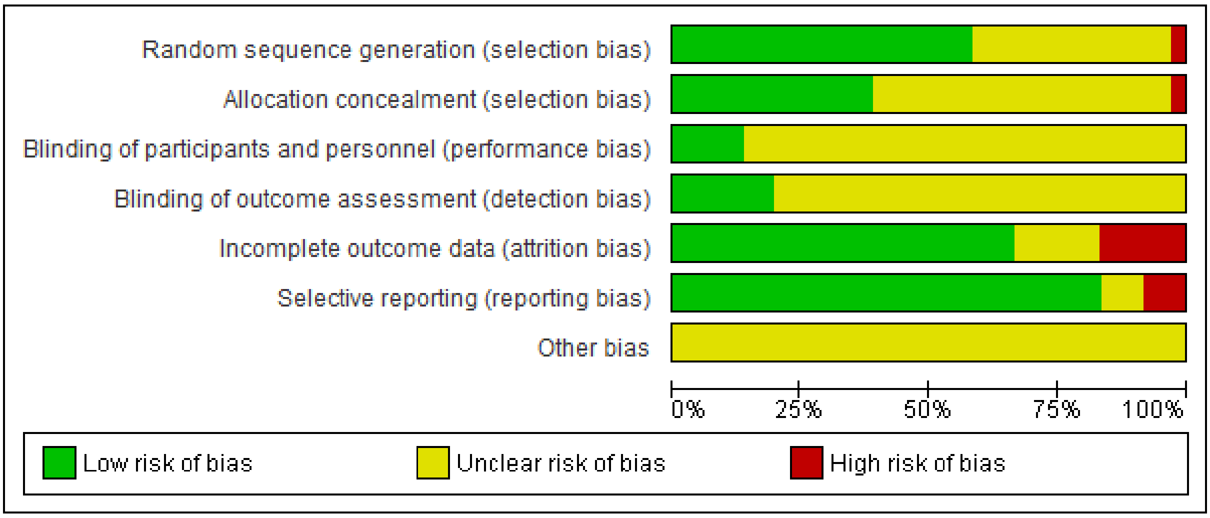

Supplementary Figure 3: Effects of telemedicine versus comparator on haemoglobin A1c at 3 and 6 months after the intervention

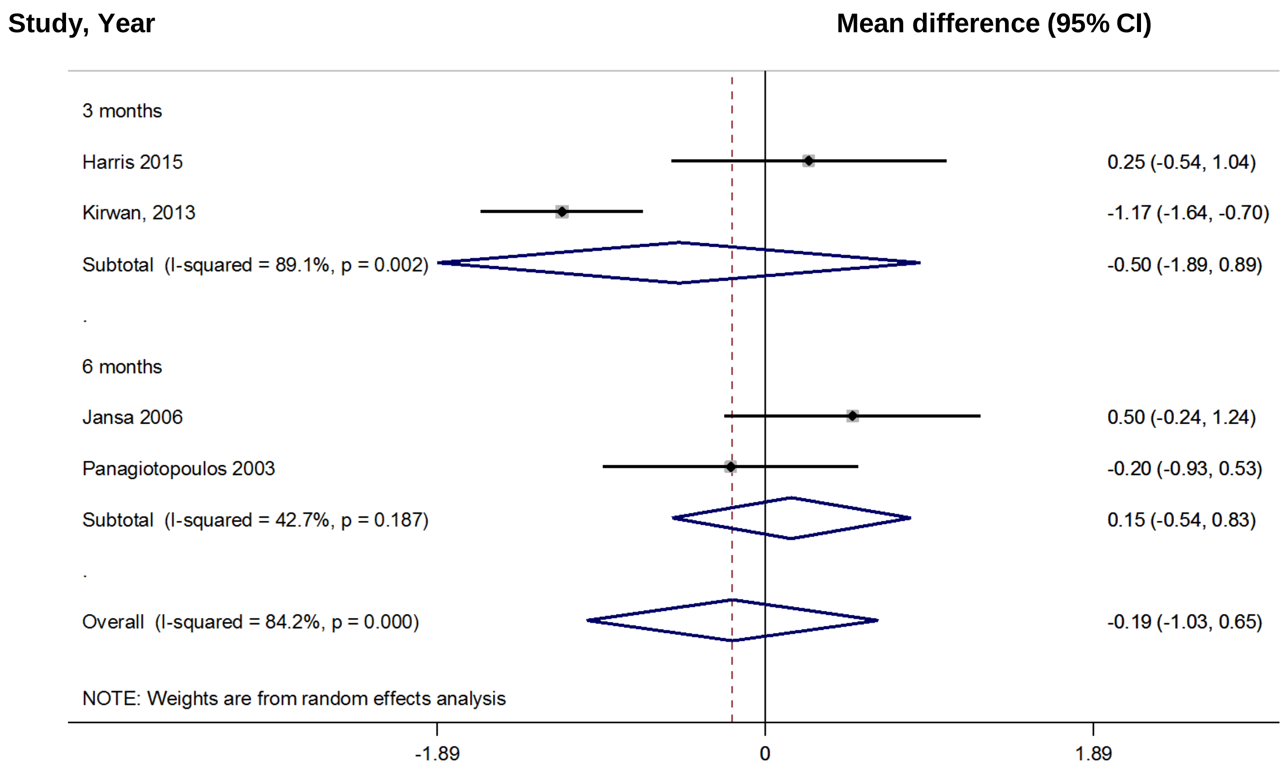

Supplementary Figure 4: Effects of telemedicine versus comparator on haemoglobin A1c, stratified by type of telemedicine

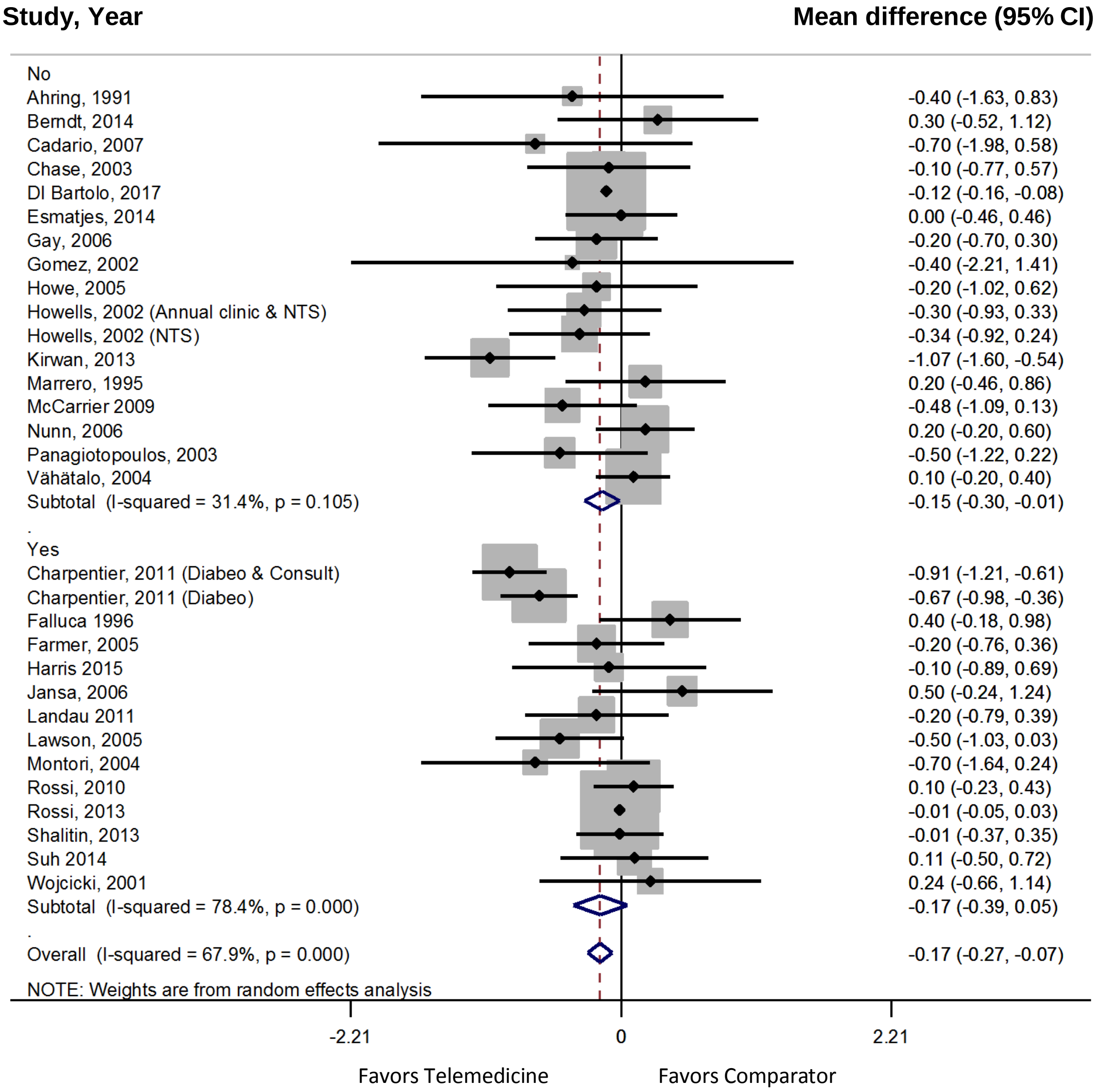

Supplementary Figure 5: Contour funnel plot using glycated haemoglobin levels at the end of intervention. The standard error of each study's effect was plotted against each trial's effect estimate. Some asymmetry was observed, with data points from 2 studies that appear to be outliers, suggesting that there was some form of publication bias but this did not reach statistical significance.

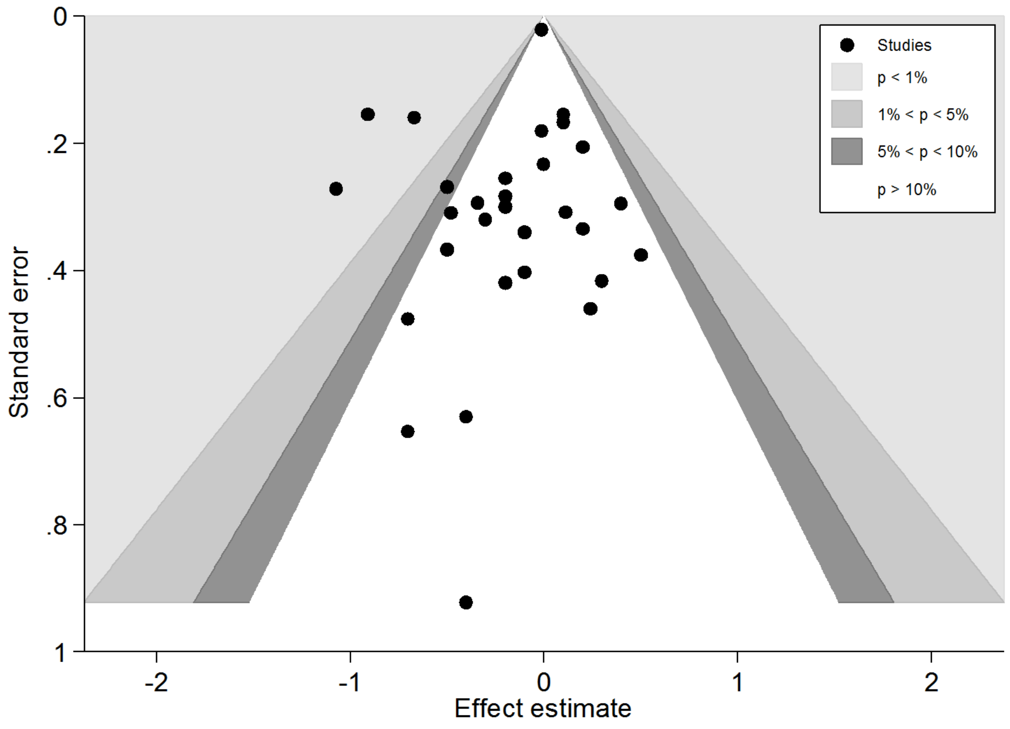

Supplementary Figure 6: Effects of telemedicine versus comparator for the change in fasting plasma glucose

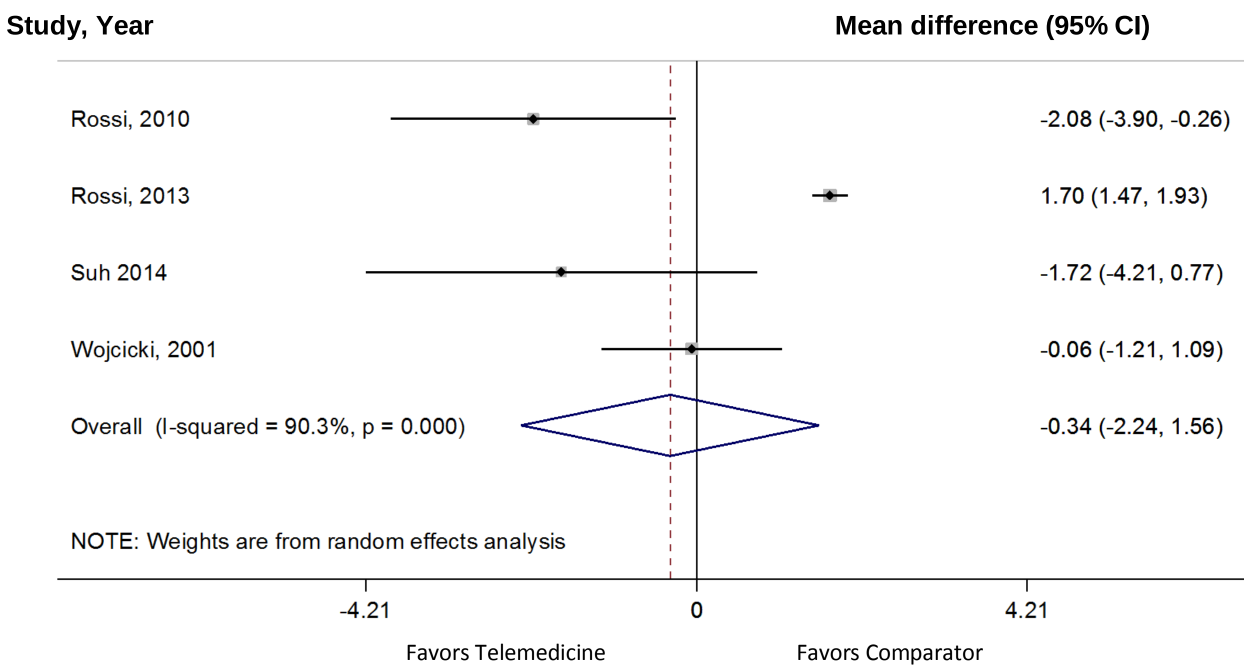

Supplementary Figure 7: Effects of telemedicine versus comparator for the change in body mass index

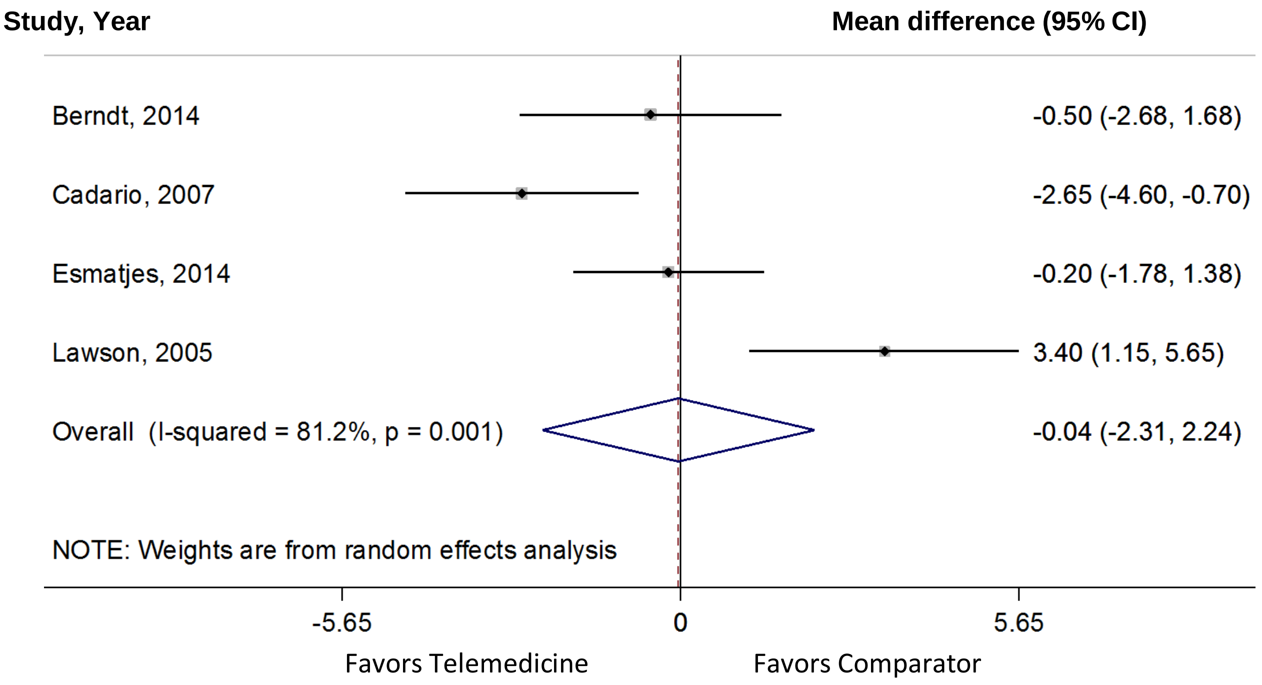

Supplementary Figure 8: Effects of telemedicine versus comparator for the change in systolic blood pressure

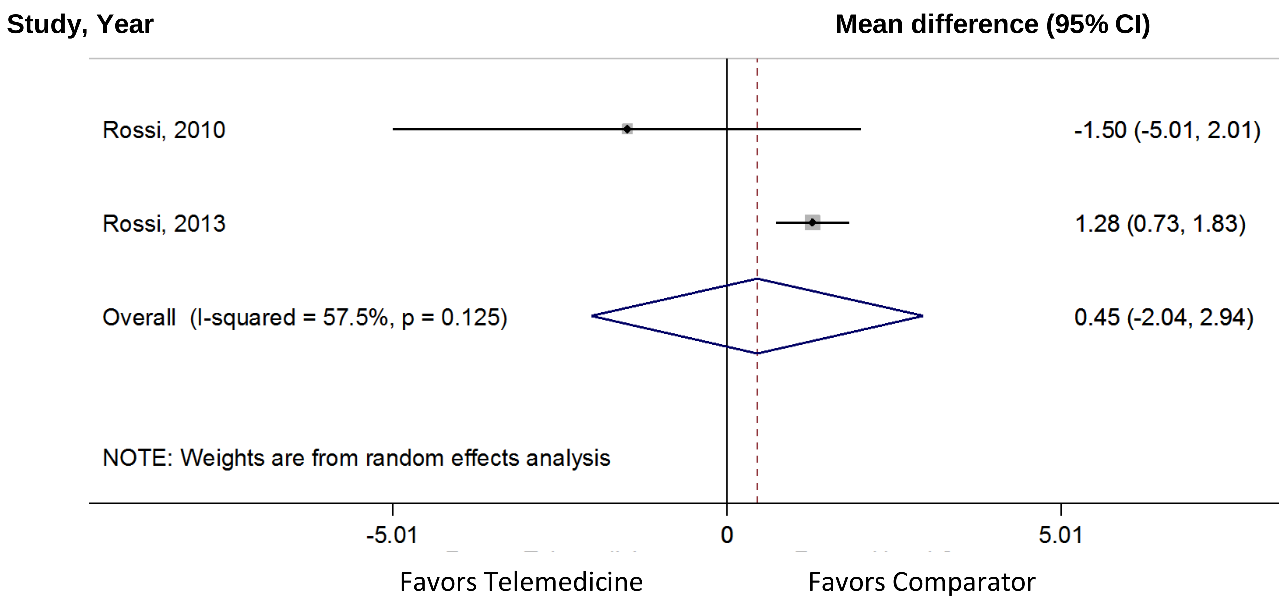

Supplementary Figure 9: Effects of telemedicine versus comparator for the change in diastolic blood pressure

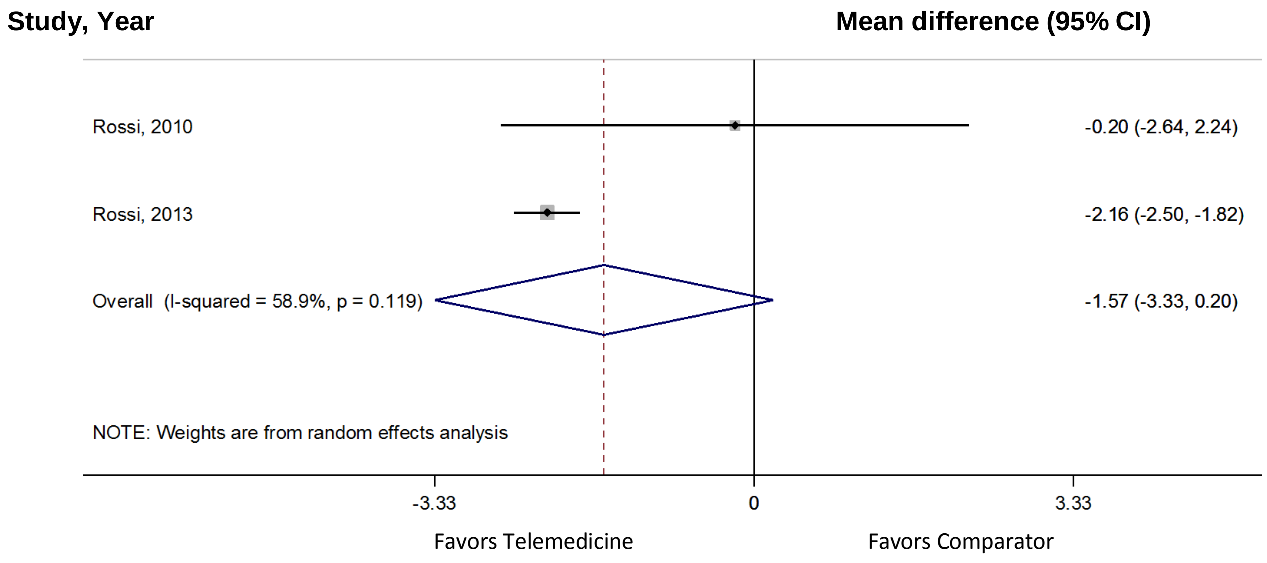

Supplementary Figure 10: Effects of telemedicine versus comparator for the change in total cholesterol

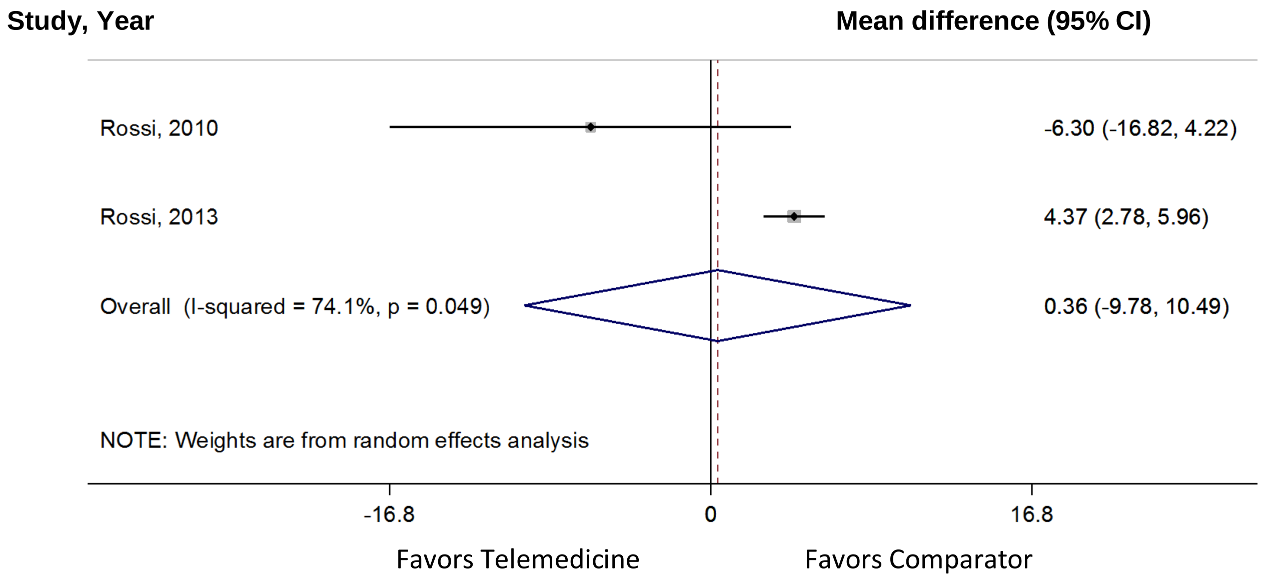

Supplementary Figure 11: Effects of telemedicine versus comparator for the change in low density lipoprotein

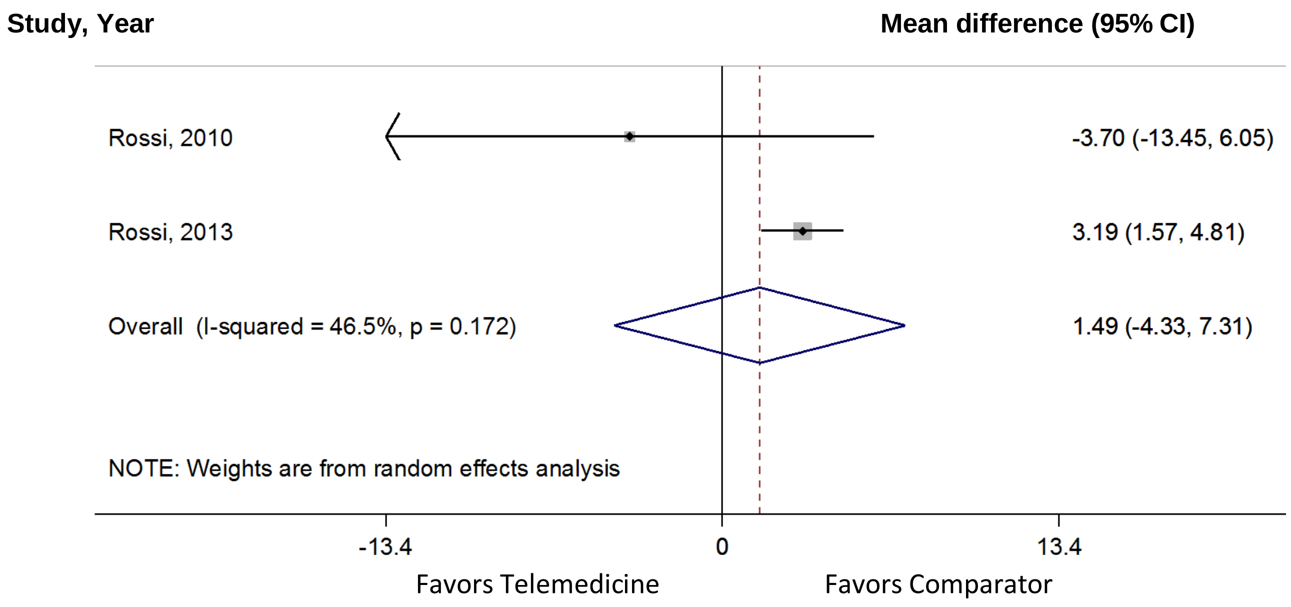

Supplementary Figure 12: Effects of telemedicine versus comparator for the change in high density lipoprotein

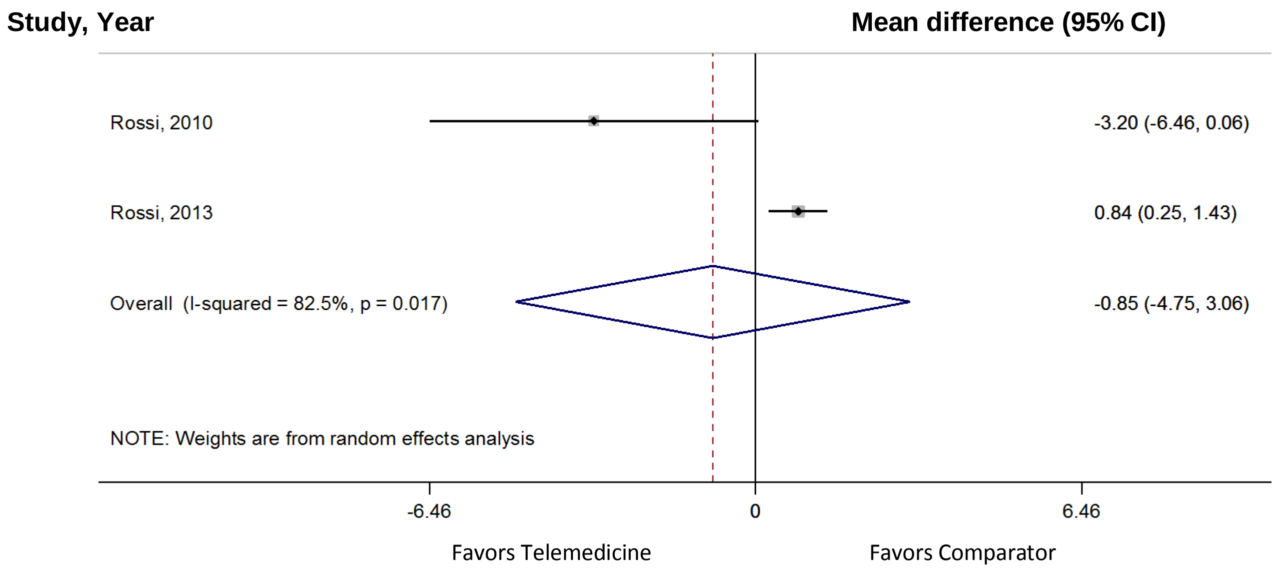

Supplementary Figure 13: Effects of telemedicine versus comparator for the change in triglyceride

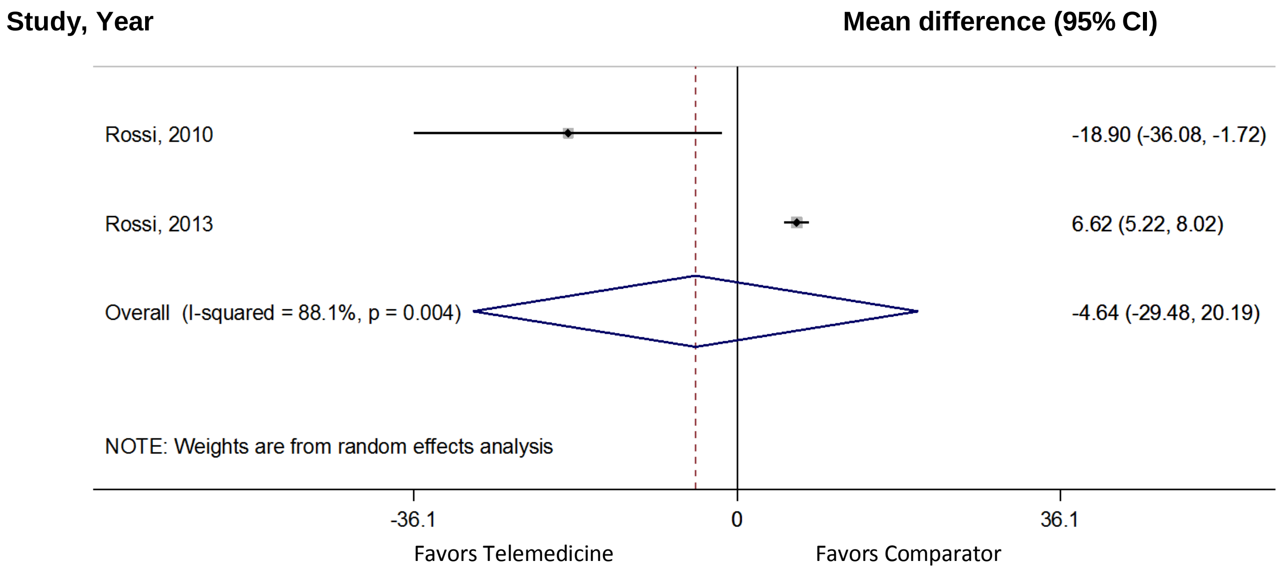

Supplementary Figure 14: Odds ratio of telemedicine intervention versus comparator on severe hypoglycemic events

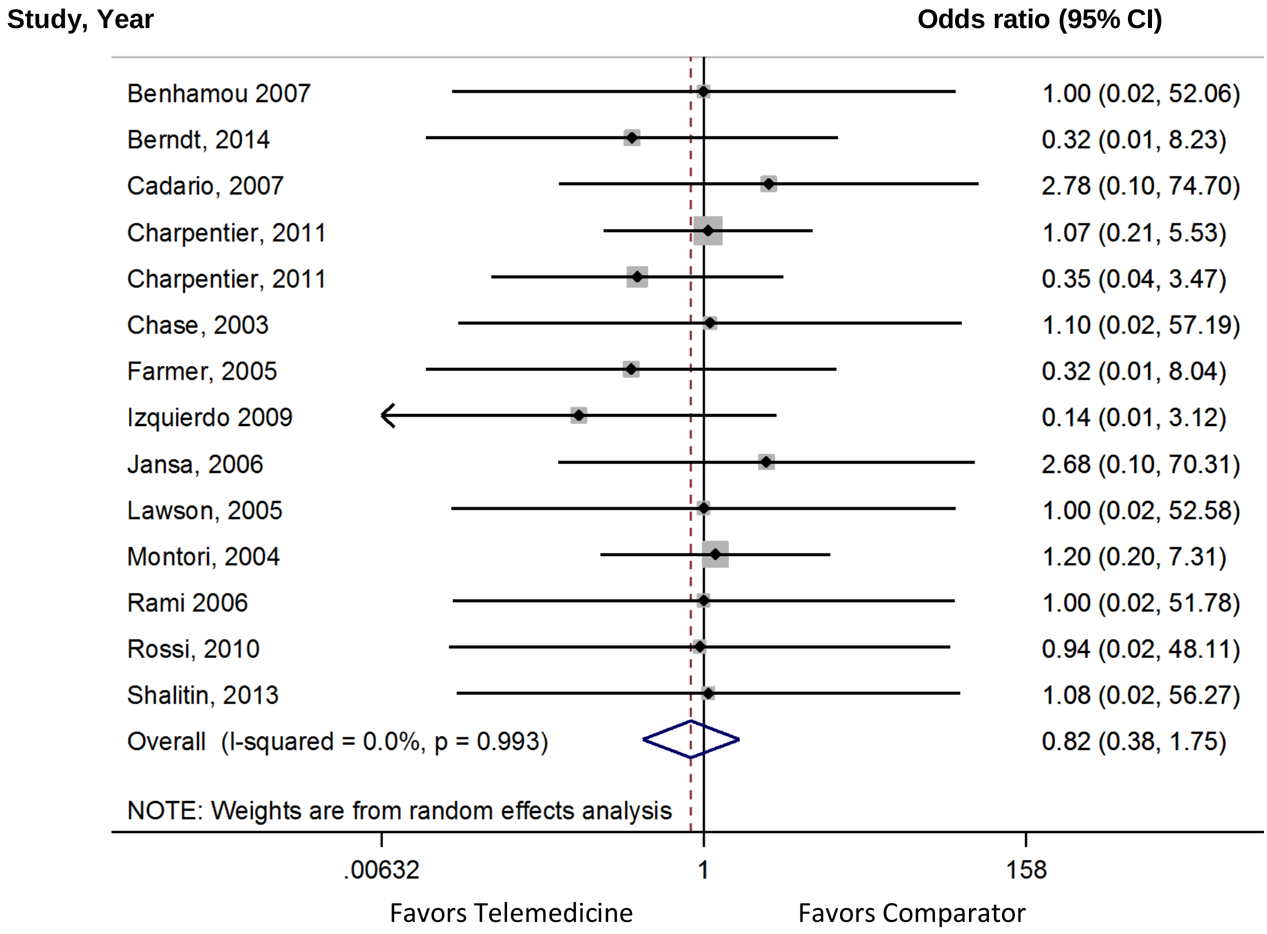

Supplementary Figure 15: Odds ratio of telemedicine intervention versus comparator on diabetic ketoacidosis events

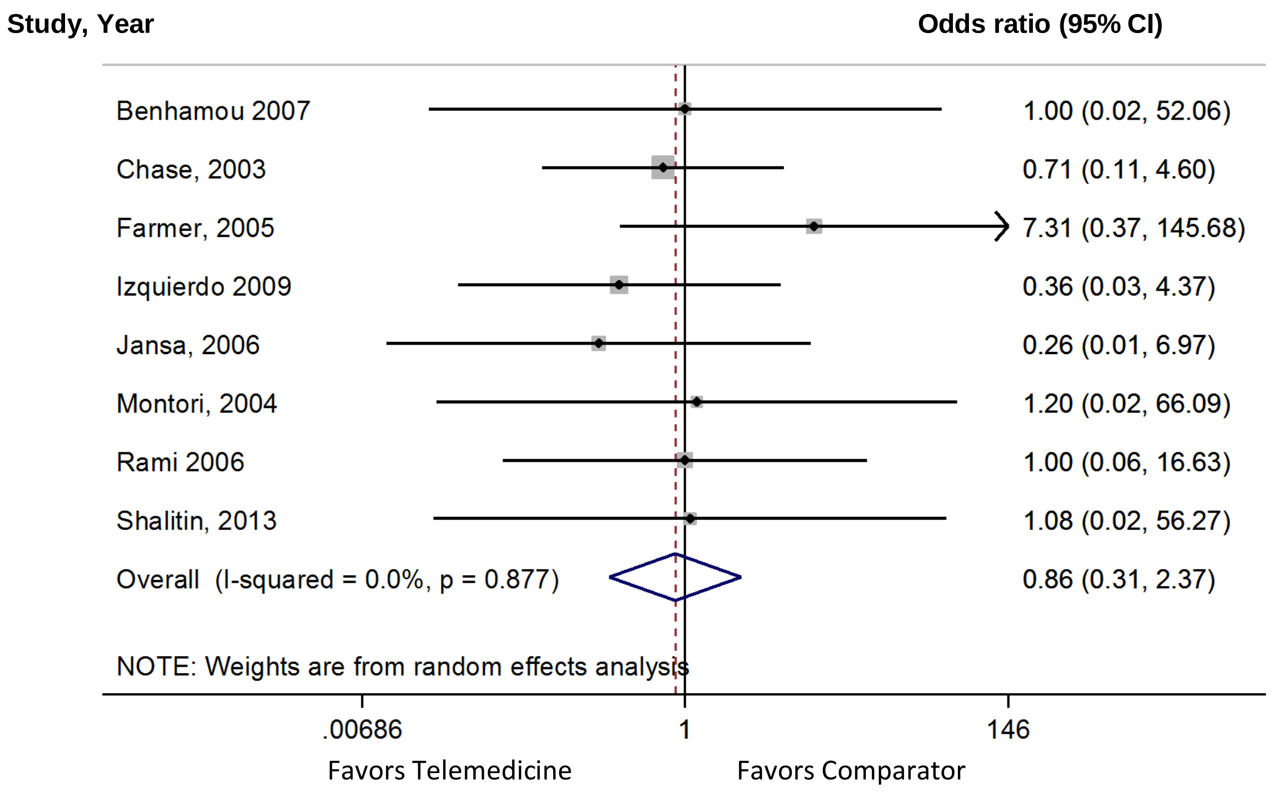

Supplementary Figure 16: Effects of telemedicine versus comparator on hemoglobin A1c in adults, stratified by type of telemedicine

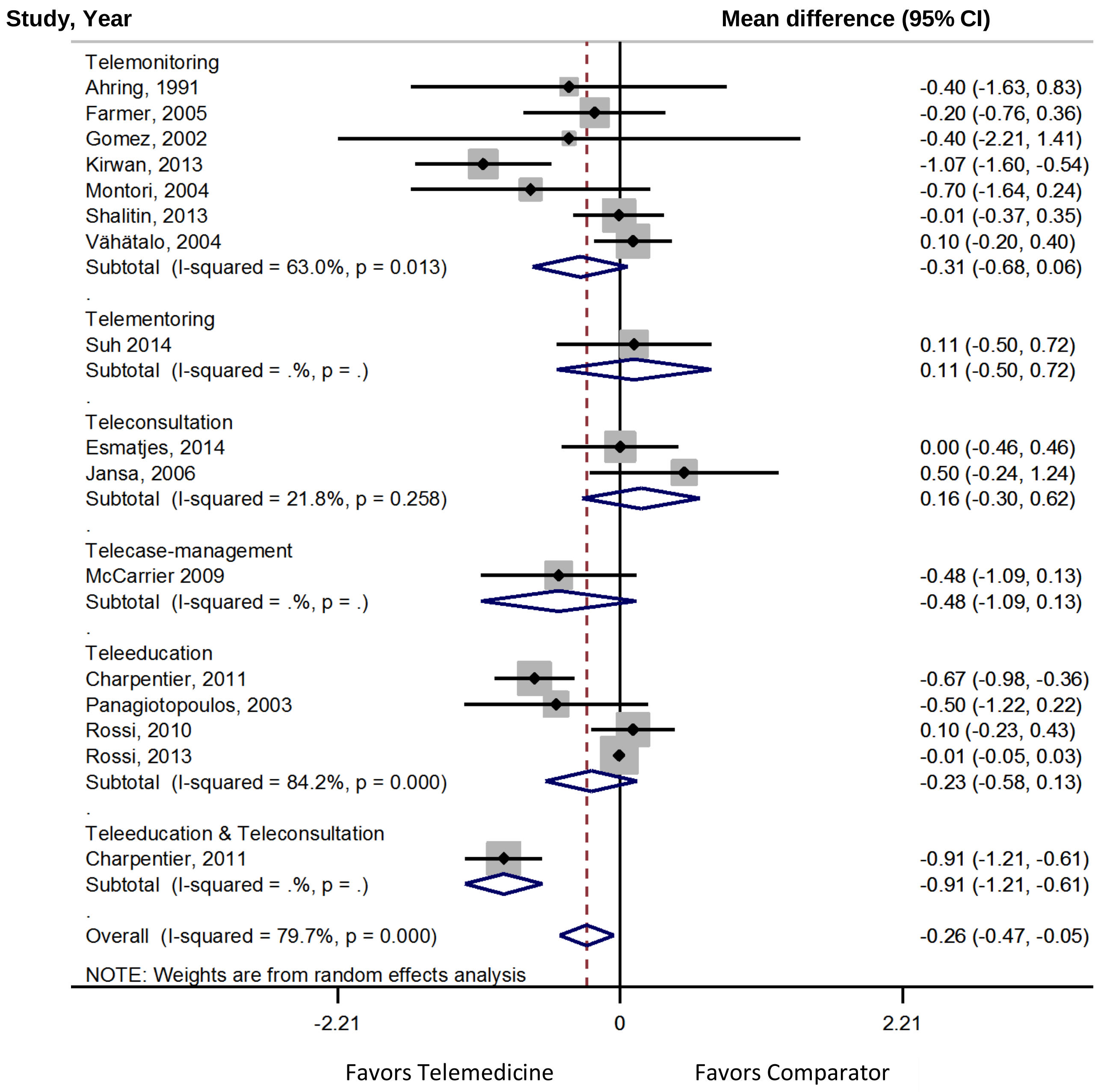

Supplementary Figure 17: Effects of telemedicine versus comparator on hemoglobin A1c in adolescents, stratified by type of telemedicine

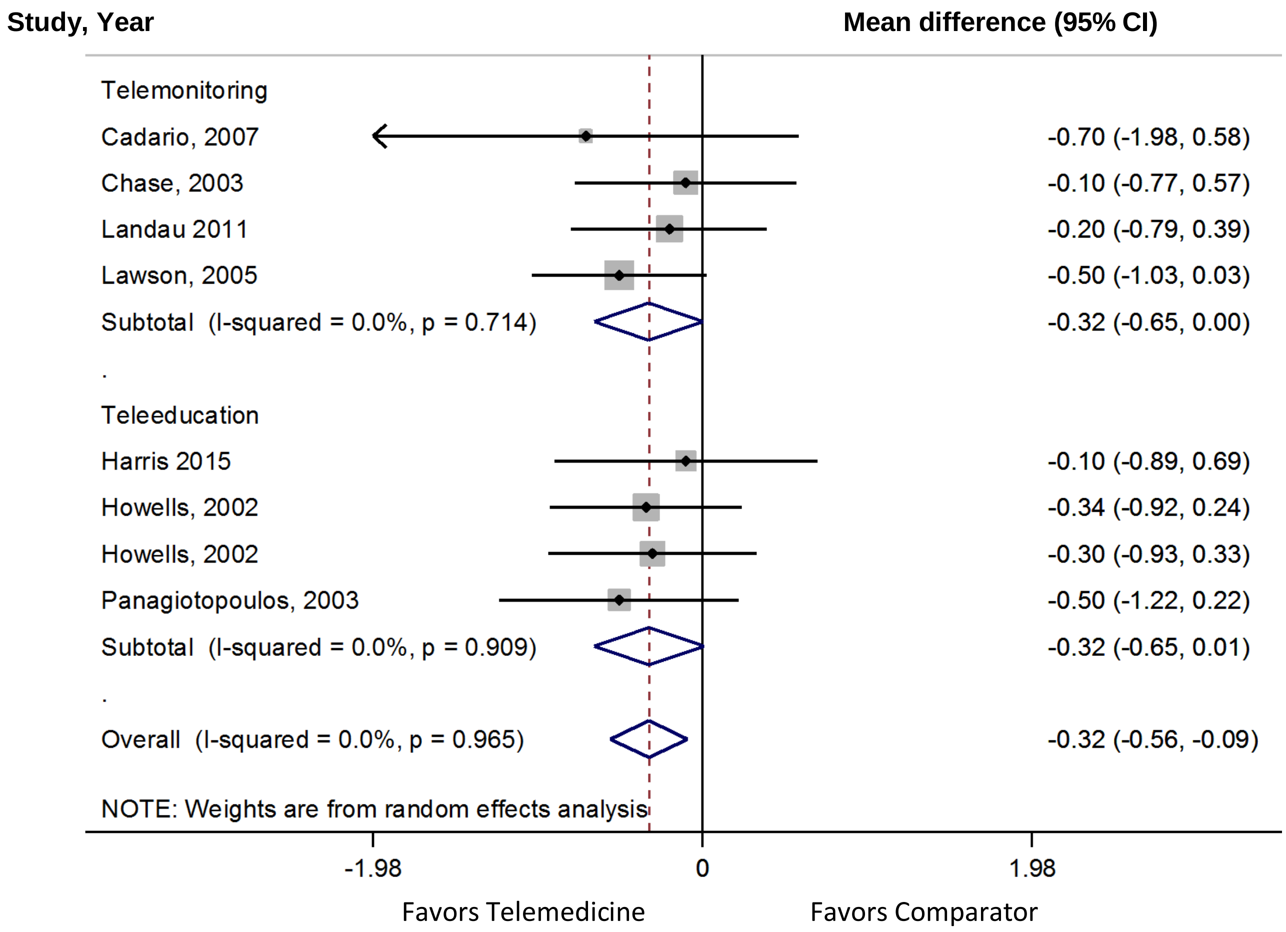

Supplementary Table 1: Selected baseline demographics and features of intervention strategies

Author, Year (Country)	Control	Intervention brief description	Sample size, n	Age (years), Diabetes duration (years)	Men, No (%)	Medium used for interventio n	Frequency of intervention and duration	Interventio n team
**Telemonitoring**								
Ahring, 1992 (Canada)	Usual care with regular clinic visit every 6 weeks	Transmission of glucose results with feedback by clinician	42	41;11.6	20 (48)	Modem, Telephone	Monitoring of blood glucose 5x/day with data transmission every week; 6 weeks	Physician
Benhamou , 2007 (France)	Usual care	Transmission of blood glucose results with feedback by clinician	30	41; 24	15 (50)	Mobile phone; Palm PDA	Weekly data download and SMS advice; 6 months	Doctor, DE
Berndt, 2014 (Germany)	Usual care	Web-based system for communication to assist in therapy and management of diabetes	68	13; 5,2	40 (59)	Internet; Smartphone	Data transmission at least 3 times a day; 4 weeks	Doctor, RN, DE, Psychologis t
Biermann, 2000 (Germany)	Usual care	Transmission of blood glucose results with revision of insulin protocols by	28	31; 9.3	NR	Modem	Data transmitted every 1-3 weeks, Consultation	Physician

		clinician.					every 2-4 weeks; 8 months	
Cadario, 2007 (Italy)	Usual care	Transmission of blood glucose results with clinician feedback	28	15; 9.1	14 (54)	Internet	Monitoring of blood glucose 4x/day with data transmission every 2 weeks; 6 months with 18 months follow up	Physician
Chase, 2003 (USA)	Usual care with regular clinic visit every 3 months	Transmission of glucose results with feedback by clinician	70	17; 7.8	30 (48)	Modem, Telephone	Data transmission every 2 weeks; 6 months	Nurse, Doctor
DeSalvo, 2014 (USA)	Continuous glucose monitoring	Remote continuous glucose monitoring (CGM)	57	14; 6.2	22 (39)	Smartphone	Daily transmission of data; 16 nights	Clinical team
Fallucca, 1996 (Italy)	Manual recording of blood glucose results and clinic visit every week	Transmission of glucose results with feedback and clinic visit every 2 weeks	17	29; 13.6	0 (0)	Modem	Data transmission weekly	Diabetologist
Farmer, 2005 (UK)	Real-time data transfer on blood glucose results with minimal feedback	Real-time data transfer on blood glucose results with intensive feedback and structured counselling	93	24; 12.5	55 (59)	Phone calls, Internet	7 minutes phone call at least fortnightly; 9 months	RN

Gomez, 2002 (Spain)	Self-monitoring of blood glucose and manual recording of results in logbook	Use of DIABTel telecare system for monitoring of data with feedback	10	32; 13.8	2 (20)	Internet; Palmtop computer	Data transmission every 2 weeks; 3 months	Doctor
Kirwan, 2013 (Australia)	Usual care with visit to physician every 3 months	Self-management application with text-message feedback and physician visit every 3 months	72	35; 18.9	28 (39)	Smartphone, SMS, Internet	Minimum of 1 weekly personalized text messages; 6 months	DE
Landau, 2012 (Israel)	Self-monitoring of blood glucose	Internet based blood glucose monitoring system with feedback	70	15; 5.9	32 (46)	Internet, Telephone	Weekly data transmission; 6 months	Dietician
Lawson, 2005 (Canada)	Usual care with education	Usual care with education, weekly standardized telephone contact with feedback by diabetic educator	46	15; 6.5	26 (57)	Telephone	Telephone contact with DNE weekly; 6 months	Doctor, RN, Dietician, DE
Marrero, 1995 (USA)	Usual care with regular clinic visit every 3 months	Transmission of glucose results with structured insulin adjustment regimen or referral	106	13; 6.2	62 (59)	Modem, Telephone	Data transmission every 2 weeks; Telephone call every 2 weeks; 3 months	Pediatric nurse practitioner

Montori, 2004 (Canada)	Usual care with transmission of blood glucose results only	Transmission of blood glucose results with clinician feedback within 24 hours	31	43; 17.1	10 (32)	Internet	Monitoring of blood glucose 4x daily and data transmission at least every 2 weeks; 6 months	Physician, RN
Rami, 2006 (Austria)	Usual care with daily written paper diary and clinic visit after 3 months	Transmission of blood glucose, carbohydrate intake and insulin dosage with feedback by clinician	36	15; 6.3	20 (56)	Mobile phone, Internet	Monitoring of blood glucose 4x/day and daily transmission of results with once weekly advice from diabetologist; 6 months	Physician
Rigla, 2007 (Spain)	Uploading of blood glucose results to a web-based communication management system (DIABTel)	Uploading of blood glucose results to a web-based communication management system (DIABTel) with physician feedback on insulin dose adjustment	10	41; 14.9	5 (50)	Internet	CGM 3 days per week; 3.5 months	Physician
Schiaffini, 2016 (Italy)	Usual care with standardised education	Standardised education and transmission of data (insulin therapy, pump	29	13; 4.3	11 (38)	Internet	Data transmission every month ; 24 months	Diabetologi st, Nurse, Dietician, Psychologis t

		setting, blood glucose data) with real-time feedback						
Shalitin, 2013 (Israel)	Regular glucose management and uploading of glucose monitoring data	Internet based blood glucose monitoring with feedback on insulin dose change by physician	70	14; 6.4	33 (47)	Internet; E-mail	Data transmission at least once a month; 4 months	Doctor
Vähätalo, 2004 (Finland)	Usual care	Transmission of blood glucose results with clinician feedback	203	43; NR	114 (56)	Telephone	Weekly first month, biweekly thereafter; 1 year	Doctor, RN
Wojcicki, 2001 (Poland)	Manual recording of self-monitoring of blood glucose results with routine clinic examination	Automated storage of blood glucose monitoring data results on e-logbook	32	26; 8.8	0 (0)	Internet, Telephone	4-9 glucose measurement daily, automatically uploaded daily; 180 days average	Diabetologist
**Teleeducation**								
Harris, 2015 (USA)	Behavioral Family System Therapy education via face-to-face	Behavioral Family System Therapy education delivered by Internet video (Skype)	90	15; 6.5	55 (61)	Internet	Up to 10 sessions that lasted 1-1.5h each; 3 months	Psychologist

Howells, 2002 (UK)	Usual care	Usual care with telephone contact to support in problem-solving	79	17; 7.0	39 (49)	Phone calls	Average of 16 calls; 12 months	Physician, Dietician
		Telephone contact to support in problem-solving, annual clinical review and 3 monthly measurement of HbA1c				Phone calls, face-to-face interaction	Average of 16 calls; 12 months	Physician, Dietician
Lehmkuhl, 2010 (USA)	Usual care	Phone calls with the study therapist reinforcing self-care activities and identification of potential barriers to management and education	32	14; NR	9 (28)	Phone calls	15-20 minutes 3 times per week telephone call; 3 months	Physician
Louch, 2013 (UK)	General health SMS text message per week	Daily SMS text message related to attitude, subjective norms perceived behavioural control and intention	19	NR	8 (39)	Telephone, SMS	Daily SMS text message; 14 days	Psychologis t
Newton, 2013 (USA)	Usual care	Interactive, website which allowed for discussion to psychosocial problems	59	15; 6.3	17 (34)	Internet	Up to 1h website visit per day; 7 weeks	Peer

Nunn, 2006 (Australia)	Usual care	Bimonthly telephone calls from pediatric diabetes educator	123	12; NR	69 (56)	Telephone	15-30 minutes bimonthly phone calls; 5-8 months	DE, Physician, RN, Dietician
Panagiotopoulos, 2003 (Canada)	Usual care	Semi-structured weekly telephone reinforcement contact from pediatric diabetes educator and review by physician	50	14; 6.3	20 (40)	Telephone	15-20 minutes call once or twice weekly;	DE, Physician
Rossi, 2010 (Italy, England, Spain)	Usual care with face-to-face education	Interactive diary with carbohydrate/ insulin bolus calculator which allows for patient physician feedback	130	36; 16.5	56 (43)	Mobile phone, SMS	Daily use of interactive diary; 6 months	Physician, Dietician
Rossi, 2013 (Italy)	Usual care with face-to-face education	Interactive diary with carbohydrate/ insulin bolus calculator which allows for patient physician feedback	127	36; 15.6	60 (47)	Mobile phone, SMS	Daily use of interactive diary; 6 months	NR
**Teleconsultation**								
Esmatjes, 2014 (Spain)	Usual hospital clinic visit	Automated uploading of blood glucose results with feedback and monthly visit by teleconference	154	32; 17.2	69 (45)	Internet	Monthly data reporting; 6 months	Physician, RN

Gay, 2006 (France)	Usual care	Reinforced follow up via telecare mediated by pharmacist in contact with hospital team	100	13; 6.2	61 (61)	Internet, Fax; Phone call	A visit to the pharmacy every 2 weeks; 6 months	Physician, Pharmacist
Izquierdo, 2009 (USA)	Usual care with clinic visit every 3 months and communication between school nurse and diabetic team as needed	Usual care with videoconferencing between school nurse, child and diabetes team every month	41	10; 4.9	NR	Internet	10-20 minutes monthly teleconference ;12 months	RN, DE
Jansa, 2006 (Spain)	Intensive clinic visit over 6 months	Telematic intensive follow-up replacing clinic visit	40	25; 11.1	21 (60)	Telephone, Telematic system	9 telematic appointments and three outpatient appointments; 6 months	RN, Physician
**Telecase-management**								
Howe, 2005 (USA)	Usual care with routine clinic visit every 3 months	Usual care and an education session on basic diabetes management skill	75	13; NR	41 (55)	Face-to-face education	One session with the study coordinator; 6 months	Doctor, RN
		Usual care, an education session on basic diabetes management skill and telephone case management				Face-to-face education, Telephone	Negotiated time; Initially weekly for 3 months, then every fortnight between 5-15	Doctor, RN

							mins; 6 months	
McCarrier, 2009 (USA)	Usual care	Usual care with web-based case management which allowed for development of individualised action plans	78	37; NR	52 (68)	Internet	Weekly email contact for feedback; 12 months	DE
**Telementoring**								
Suh, 2014 (Korea)	Transmission of glucose results to website	Transmission of glucose results to website with individualized feedback from mentors	57	32; 7.4	21 (37)	Telephone	4 times daily, 7 days a week; data transmission every 2 weeks with feedback from mentor	Peer
**Teleeducation & Teleconsultation**								
Charpentie r, 2011 (France)	Usual care with paper logbook monitoring	Interactive online diary (Diabeo software) installed in smartphone	180	34; 16.4	66 (37)	Smartphone	Daily use of software; 6 months	Physician
		Interactive online diary (Diabeo software) installed in smartphone and				Smartphone, Phone calls	Daily use of software, teleconsultatio nevery 2	Physician

		teleconsultations					weeks; 6 months	

NR- Not reported, SMS – Short messaging system, RN – Registered nurse, DE- Diabetes educator, DNE – Diabetes nurse educator

Supplementary Table 2: Key intervention component of studies included in the current review

Author	Intervention									Control								
	Individualised care plan	Audit & Feedback	Psychology based counselling	Skill building	Financial incentive	Problem solving	Peer / Family support	Theory based counselling	Lay health advisor	Individualised care plan	Audit & Feedback	Psychology based counselling	Skill building	Financial incentive	Problem solving	Peer / Family support	Theory based counselling	Lay health advisor
**Articles examining teleeducation**																		
Harris, 2015			Y			Y	Y	Y										
Howells, 2002 – Usual care with telephone support			Y			Y		Y									Y	
Howells, 2002 – Usual care with support and regular clinic visit			Y			Y		Y									Y	
Lehmkuhl, 2010			Y	Y		Y		Y										
Louch, 2013			Y														Y	
Newton, 2013		Y		Y		Y		Y										
Nunn, 2006								Y										
Panagiotopoulos, 2003	Y	Y		Y				Y										
Rossi, 2010	Y	Y		Y		Y												

Rossi, 2013	Y	Y		Y		Y											Y	
**Articles examining telemonitoring**																		
Ahring, 1992	Y	Y																
Benhamou, 2007		Y																
Berndt, 2014		Y																
Biermann, 2000				Y		Y							Y					
Cadario, 2007		Y																
Chase, 2003	Y	Y																
DeSalvo, 2014		Y									Y							
Fallucca, 1996				Y														
Farmer, 2005		Y									Y							
Gomez, 2002	Y	Y				Y												
Kirwan, 2013		Y						Y										
Landau, 2012	Y	Y		Y									Y					
Lawson, 2005		Y		Y	Y		Y											
Marrero, 1995		Y	Y															
Montori, 2004		Y																
Rami, 2006		Y				Y												
Rigla, 2007		Y																
Schiaffini, 2016		Y		Y									Y					
Shalitin, 2013		Y									Y							
Vähätalo, 2004		Y																
Wojcicki, 2001		Y									Y							
**Articles examining teleconsultation**																		
Esmatjes, 2014	Y	Y																
Gay, 2006	Y	Y							Y									
Izquierdo, 2009	Y	Y		Y														
Jansa, 2006		Y				Y					Y				Y			

**Articles examining telecase-management**																			
Howe, 2005	Usual care with education	Y	Y		Y						Y								
	Usual care, education and case-management	Y	Y		Y		Y		Y		Y								
McCarrier, 2009		Y	Y		Y		Y												
**Articles examining telementoring**																			
Suh, 2014		Y	Y		Y			Y		Y	Y			Y					
**Articles examining teleeducation & teleconsultation**																			
Charpentier, 2011	Interactive diary	Y	Y		Y									Y					
	Interactive diary with teleconsultation	Y	Y		Y														

Supplementary Table 3: Details of educational content delivered to participants in studies examining teleeducation

Author		Intervention						Control					
		**Diabetes management**	**Diet**	**Diabetes complications (foot care, retinopathy)**	**Diabetes symptoms (e.g. hypoglycemia)**	**Medication adherence**	**Education trivia / challenge**	**Diabetes management**	**Diet**	**Diabetes complications (foot care, retinopathy)**	**Diabetes symptoms (e.g. hypoglycemia)**	**Medication adherence**	**Education trivia / challenge**
**Articles examining teleeducation**													
Harris, 2015		Y											
Howells, 2002	Usual care with telephone support	Y						Y					
	Usual care with support and regular clinic visit	Y						Y					
Lehmkuhl, 2010		Y	Y	Y	Y								
Louch, 2013		Y		Y				Y					
Newton, 2013		Y	Y	Y	Y								
Nunn, 2006		Y	Y	Y	Y								
Panagiotopoulos, 2003		Y		Y				Y					
Rossi, 2010		Y	Y										
Rossi, 2013		Y	Y					Y					
**Articles examining teleeducation & teleconsultation**													
Charpentier, 2011	Interactive	Y	Y										

	diary												
	Interactive diary with teleconsultation	Y	Y										

Supplementary Table 4: Details of self-monitoring frequency and recommended schedule

Author	Intervention				Control			
	**Monitoring frequency (Target readings per week)**	**SMBG Schedule**	**No of days over which monitoring is spread**	**Person delivering meter training**	**Monitoring frequency (Target readings per week)**	**SMBG Schedule**	**No of days over which monitoring is spread**	**Person delivering meter training**
**Articles examining telemonitoring**								
Ahring, 1992	Five readings daily	Before breakfast, before lunch, afternoon, before dinner and bedtime	Twice/week					
Benhamou, 2007								
Berndt, 2014	Minimum 3 times daily							
Biermann, 2000								
Cadario, 2007	4 times per day							
Chase, 2003	Variable depending on meter results							

DeSalvo, 2014	Continuous glucose monitoring	Continuous glucose monitoring	Continuous glucose monitoring		Continuous glucose monitoring	Continuous glucose monitoring	Continuous glucose monitoring	
Fallucca, 1996	Minimum 3 times daily	Before and after breakfast, lunch and dinner						
Farmer, 2005								
Gomez, 2002								
Kirwan, 2013								
Landau, 2012	Reporting SMBG weekly			Study coordinator				
Lawson, 2005	3 times daily (breakfast, supper and bedtime) and lunch time on weekends							
Marrero, 1995								
Montori, 2004	4 times daily	7 days a week	Throughout the week					
Rami, 2006								
Rigla, 2007	Minimum twice a day							

Schiaffini, 2016								
Shalitin, 2013	Minimum once a month data upload			Physician				
Vähätalo, 2004	Up to 25 test per week			Diabetes nurse	Up to 10 test per week			
Wojcicki, 2001	Six readings daily	Pre and 60 mins post meal	Daily					

Supplementary Table 5: Details of the action(s) taken based upon results from glucose monitoring

Author	Intervention				Control			
	**Action taken according to SMBG results**	**Parameter / Trigger for action**	**Interventionist**	**Area of changes made**	**Action taken according to SMBG results**	**Parameter / Trigger for action**	**Interventionist**	**Area of changes made**
**Articles examining telemonitoring**								
Ahring, 1992	Counselling /Feedback upon 1week of receipt of results			Insulin dosage or food intake advice				
Benhamou, 2007								
Berndt, 2014								
Biermann, 2000								
Cadario, 2007	Feedback within 2-3 days upon receipt of results every 2 weeks		Clinician					
Chase, 2003								
DeSalvo, 2014	Treatment for confirmed hypoglycae	Less than 70mg/dL	Medical personel		Audible alarm to alert patient for self-	Less than 70mg/dL		

	mia				treatment			
Fallucca, 1996								
Farmer, 2005	Immediate graphical feedback with colour codings		Diabetes specialist nurse	Telephone to address any areas of concerns and problems	Graphical feedback for previous 24hrs			
Gomez, 2002	Feedback within 24 hours of receipt of results at least once every 2 weeks		Doctor	therapy adjustment, diet plan modification or additional information request				
Kirwan, 2013								
Landau, 2012	Phone call to recommend changes for treatment after consultation with physician	Poor glycaemic control	Study coordinator	Insulin dose				
Lawson, 2005	Phone call to discuss results	Any readings below 4mmol/L or higher than 8mmol/L	Diabetes doctor or nurse	Insulin dose adjustment				
Marrero, 1995	Feedback and review	No more than 2	Pediatric nurse	Insulin dosage				

	every 2 weeks	values <80mg/dL within a week	practitioner	adjustment, clinic visit, dietary service referral					
Montori, 2004	Feedback within 24 hours		Study nurse supervised by endocrinolo gist						
Rami, 2006	Automated generated SMS feedback or personalised message		Diabetologis t						
Rigla, 2007									
Schiaffini, 2016									
Shalitin, 2013			Physician	Insulin dose adjustment			Physician	Insulin dose adjustment
Vähätalo, 2004	Feedback regardless of results		Doctor						
Wojcicki, 2001									

Supplementary Table 6: Analysis of study intervention features

Feature	No of studies		Treatment effects (95% CI)	
	With feature	Without feature	With feature	Without feature
**Key component of intervention**				
Individualised assessment: patient is assessed individually and intervention is tailored to the intervention	13	15	-0.27 (-0.49 to -0.04)	-0.10 (-0.31 to 0.11)
Audit & feedback: providing feedback based upon results to patients on their diabetes control or performance	24	4	-0.22 (-0.38 to -0.06)	0.01 (-0.27 to -0.30)
Psychological based counselling: intervention to modify behaviour, belief or perception of the patient regarding their diabetes	3	25	-0.16 (-0.48 to 0.17)	-0.19 (-0.35 to -0.03)
Skill building: developing skills, diabetes knowledge and patients' ability to self-manage their diabetes	10	18	-0.27 (-0.54 to 0.00)	-0.10 (-0.27 to 0.06)
Problem solving: development of patient's problem solving skill required to manage their diabetes	8	20	-0.01 (-0.05 to 0.03)	-0.22 (-0.42 to -0.01)
Theory based counselling: educational intervention (including self-management, monitoring or behaviour-related task)	5	23	-0.30 (-0.71 to 0.11)	-0.16 (-0.31 to 0.00)
**Intervention characteristics**				
High intensity: the patient is in direct contact with intervention at least once weekly	13	14	-0.24 (-0.49 to 0.01)	-0.09 (-0.23 to 0.06)
Duration of study: intervention is delivered for at least 6 months	21	7	-0.24 (-0.41 to -0.07)	0.07 (-0.16 to 0.31)
Multifaceted intervention: intervention has at least 3 or more key component	12	16	-0.32 (-0.55 to -0.08)	-0.05 (-0.24 to 0.15)
Number of interventionist: intervention has 3 or more different providers as part of the intervention team	3	25	-0.02 (-0.53 to 0.48)	-0.22 (-0.38 to -0.04)
Active control : control group had some form of intervention beyond usual care but not meeting our operational definition of	13	15	-0.19 (-0.37 to 0.05)	-0.18 (-0.38 to 0.01)

telemedicine				
**Study baseline characteristics**				
High baseline HbA1c: patients recruited into the study had a median HbA1c ≥9%	12	16	-0.34 (-0.57 to -0.11)	-0.06 (-0.20 to 0.09)
Study continent: study was conducted in North America	9	19	--0.30 (-0.54 to -0.06)	-0.15 (-0.32 to 0.02)

Supplementary Table 7: Results of meta-analysis of previously conducted studies compared to current study

**Systematic review study**	**Inclusion criteria**	**No of trials**	**No of participants**	**Treatment effects (95% CI)**
Current study	Trials examining telemedicine in all patients, either whole trial population or a subgroup reported separately	26	2013	HbA1c MD:-0.18% (-0.33 to -0.03)<br>Severe hypoglycaemia OR: 0.82 (0.35 to 1.75)<br>Diabetic ketoacidosis OR: 0.86 (0.31 to 2.37)
Montori, 2004	Trials of telecare versus usual care or other forms of data transmission with or without feedback	8	355	HbA1c MD: -0.21% (-0.62 to 0.20)
Schulman 2010	Trials in youth aged less than 19 years that involved routine transmission of blood glucose with clinician feedback	9	568	HbA1c MD: -0.12% (-0.35 to 0.11)<br>Severe hypoglycaemia OR: 1.42 (0.22 to 9.32)<br>Diabetic ketoacidosis OR: 1.02 (0.24 to 4.23)
Marcolino, 2013	Trials examining any telemedicine application in adult care (≥18 years) in patients with diabetes (Type 1 & Type 2).	2	145	HbA1c* MD: -0.86% (-1.12 to -0.59)
Hou, 2016	Trials examining telehealth programs using diabetes apps in adults (>18 years) with diabetes (Type 1 & Type 2)	4	426	HbA1c* MD: -0.36% (-0.87 to 0.14)

* Results presented are for the subgroup analysis including only type 1 diabetes patients.

CI: Confidence interval; MD: Mean difference; OR: Odds ratio

References:

- Farmer AO, Gibson OJ, Tarassenko L, Neil A. A systematic review of telemedicine interventions to support blood glucose self-monitoring in diabetes. Diabetic Medicine. 22(10):1372-8. 2005

- Hou C, Carter B, Hewitt J, Francisa T, Mayor S. Do mobile phone applications improve glycemic control (HbA1c) in the self-management of diabetes? A systematic review, meta-analysis and GRADE of 14 randomised trials. Diabetes Care. 2016 39(11): 2089-2095. doi:10.2337/dc16-0346

- Montori VM, Helgemoe PK, Guyatt GH, Dean DS, Leung TW, Smith SA, Kudva YC. Telecare for Patients With Type 1 Diabetes and Inadequate Glycemic Control A randomized controlled trial and meta-analysis. Diabetes Care. 27(5):1088-94. 2004
- Su D, Zhou J, Kelley MS, Michaud TL, Siahpush M, Kim J, Wilson F, Stimpson JP, Pagán JA. Does telemedicine improve treatment outcome for diabetes? A meta-analysis of results from 55 randomised controlled trials. Diabetes Research and Clinical Practice. 116: 136-48. 2016
- Schulman RM, O'Gorman CS, Palmert MR. The impact of telemedicine interventions involving routine transmission of blood glucose data with clinician feedback on metabolic control in youth with type 1 diabetes: A systematic review and meta—analysis. International Journal of Pediatric Endocrinology. 2010. Doi: 10.1155/2010/536957.

Supplementary Table 8: Quality of included studies, assessed using the GRADE system

Quality assessment							№ of patients		Effect		Quality
**№ of studies**	**Study design**	**Risk of bias**	**Inconsistency**	**Indirectness**	**Imprecision**	**Other considerations**	**Telemedicine**	**usual care**	**Relative (95% CI)**	**Absolute (95% CI)**	
**HbA1c - End of intervention**											
28	randomised trials	serious [a]	serious [a]	serious [b]	not serious	none	1084	1015	-	MD **0.18 % lower** (0.33 lower to 0.03 lower)	⊕◯◯◯ VERY LOW
HbA1c - 3mo follow up (follow up: mean 3 months)											
2	randomised trials	serious [c]	serious [a]	serious [b]	not serious	publication bias strongly suspected [d]	71	72	-	MD **0.5 % lower** (1.89 lower to 0.89 higher)	⊕◯◯◯ VERY LOW
HbA1c - 6mo follow up (follow up: mean 6 months)											

Quality assessment							№ of patients		Effect		Quality
**№ of studies**	**Study design**	**Risk of bias**	**Inconsistency**	**Indirectness**	**Imprecision**	**Other considerations**	**Telemedicine**	**usual care**	**Relative (95% CI)**	**Absolute (95% CI)**	
2	randomised trials	serious [c]	serious [a]	serious [b]	not serious	publication bias strongly suspected [d]	71	72	-	MD **0.5 % lower** (1.89 lower to 0.89 higher)	⨁◯◯◯ VERY LOW
Fasting plasma glucose											
4	randomised trials	serious [d]	serious [a]	serious [b]	not serious	none	162	162	-	MD **0.34 mmol/L lower** (2.24 lower to 1.56 higher)	⨁◯◯◯ VERY LOW
Body mass index											

Quality assessment							№ of patients		Effect		Quality
№ of studies	Study design	Risk of bias	Inconsistency	Indirectness	Imprecision	Other considerations	Telemedicine	usual care	Relative (95% CI)	Absolute (95% CI)	
4	randomised trials	serious [c]	serious [a]	serious [b]	serious [c]	none	125	133	-	MD **0.04 kg/m2 lower** (2.31 lower to 2.24 higher)	⊕◯◯◯ VERY LOW
Systolic blood pressure											
2	randomised trials	serious [c]	serious [e]	serious [b]	very serious [c]	none	122	120	-	MD **0.45 mmHg higher** (2.04 lower to 2.94 higher)	⊕◯◯◯ VERY LOW
Diastolic blood pressure											

Quality assessment							№ of patients		Effect		Quality
**№ of studies**	**Study design**	**Risk of bias**	**Inconsistency**	**Indirectness**	**Imprecision**	**Other considerations**	**Telemedicine**	**usual care**	**Relative (95% CI)**	**Absolute (95% CI)**	
2	randomised trials	serious [c]	serious [e]	serious [b]	very serious [c]	none	122	120	-	MD **1.57 mmHg lower** (3.33 lower to 0.2 higher)	⊕◯◯◯ VERY LOW
Total cholesterol											
2	randomised trials	serious [c]	serious [e]	serious [b]	very serious [c]	none	122	120	-	MD **0.36 mmol/L higher** (9.78 lower to 10.49 higher)	⊕◯◯◯ VERY LOW
Low-density lipoprotein											

Quality assessment							№ of patients		Effect		Quality
**№ of studies**	**Study design**	**Risk of bias**	**Inconsistency**	**Indirectness**	**Imprecision**	**Other considerations**	**Telemedicine**	**usual care**	**Relative (95% CI)**	**Absolute (95% CI)**	
2	randomised trials	serious [c]	serious [e]	serious [b]	very serious [c]	none	122	120	-	MD **1.49 mmol/L higher** (4.33 lower to 7.31 higher)	⨁◯◯◯ VERY LOW
High density lipoprotein											
2	randomised trials	serious [c]	serious [e]	serious [b]	very serious [c]	none	122	120	-	MD **0.85 mmol/L lower** (4.75 lower to 3.06 higher)	⨁◯◯◯ VERY LOW
Triglycerides											

Quality assessment							№ of patients		Effect		Quality
**№ of studies**	**Study design**	**Risk of bias**	**Inconsistency**	**Indirectness**	**Imprecision**	**Other considerations**	**Telemedicine**	**usual care**	**Relative (95% CI)**	**Absolute (95% CI)**	
2	randomised trials	serious [c]	serious [e]	serious [b]	very serious [c]	none	122	120	-	MD **4.64 mmol/L lower** (29.48 lower to 20.19 higher)	⊕◯◯◯ VERY LOW
Severe hypoglycaemia											
13	randomised trials	serious [f]	not serious	not serious	serious [g]	none	9/484 (1.9%)	10/421 (2.4%)	**OR 0.82** (0.35 to 1.75)	**4 fewer per 1,000** (from 15 fewer to 17 more)	⊕⊕◯◯ LOW
Diabetic ketoacidosis											

Quality assessment							№ of patients		Effect		Quality
**№ of studies**	**Study design**	**Risk of bias**	**Inconsistency**	**Indirectness**	**Imprecision**	**Other considerations**	**Telemedicine**	**usual care**	**Relative (95% CI)**	**Absolute (95% CI)**	
8	randomised trials	serious [d,f]	not serious	not serious	serious [c,g]	none	7/221 (3.2%)	7/219 (3.2%)	**OR 0.86** (0.31 to 2.37)	**4 fewer per 1,000** (from 22 fewer to 41 more)	⊕⊕◯◯ LOW

CI: Confidence interval; **MD:** Mean difference; **OR:** Odds ratio

a. High level of heterogeneity was observed despite performing subgroup analysis
b. Surrogate marker
c. Very small sample size and lack of reporting of outcome
d. Selective outcome reporting
e. Large variation in effect
f. Lack of blinding
g. Large confidence interval and few events
